# Supplementary material for: “They Reckon They’re Man’s Best Friend and I Believe That.” Understanding Relationships with Dogs in Australian Aboriginal Communities to Inform Effective Dog Population Management
Source: Animals (Basel). 2020 May 7;10(5):810. doi: 10.3390/ani10050810 (PMC7278576; doi:10.3390/ani10050810)
Supplement: Supplementary file 1 [file animals-10-00810-s001.pdf]

Dog Owner Survey

This study is about pet ownership in remote communities in New South Wales. The study involves completing a survey and is being done by The University of Sydney with the RSPCA and NSW Health Aboriginal Environmental Health Unit.

The study aims to 1) better understand Aboriginal people's relationships with their dogs and cats; 2) to understand the effects of an RSPCA Community Companion Animal Health Program (dog program) and how the dog program might be made better; 3) to see if community housing in NSW supports healthy pet ownership (e.g. condition of fences)

You have been invited to do this survey because you have pet dogs and/or cats and you live in NSW.

You will be given an information statement to keep with more information about the study. If you have any more questions you can contact Gemma Ma (gemm.ma@sydney.edu.au).

Participation in this research study is voluntary. If you decide you want to be in the study then change your mind later, that's ok. All you need to do is tell us that you don't want to be in the study anymore.

Submitting the completed survey is an indication of your consent to participate in the study. By giving your consent to take part in this study you are telling us that you understand what you have read, agree to take part in the research study and agree to the use of your personal information as described in the information statement.

If you have any complaints or concerns about the study you can Call the university on +61 2 8627 8176 or Write an email to human.ethics@sydney.edu.au

CONTACT INFORMATION (this information will be stored separate from your survey responses so no one will know what your answers were)

Name: ______________________________

Address: ______________________________

Would you like to receive feedback about the overall results of the study? (Feedback will be in the form of a one-page summary when the study is finished)

◯ Yes
◯ No

SECTION 1 - Your relationship with your dog

Do you identify as Aboriginal or Torres Strait Islander?

◯ Yes
◯ No

Tell us about your dogs

(We would love to know how many dogs and how many cats, if they are boys or girls, if they are desexed or if they've had any litters, where you got them and how long they have lived with you e.g. Missy is a mastiff dog, she's 3 years old, she's had two litters, her last puppies are 3 weeks old. We got her from a friend as a pup)

6. Have you used the RSPCA Community Companion Animal Health Program (the "dog program") before (since 2015)

◯ Yes
◯ No
◯ Unsure

If yes, what was your pet's name?

____

If yes, where is the animal now?
(You can pick more than one option if applicable)

☐ Still living with owner
☐ Rehomed
☐ Died
☐ Don't know
☐ Other

Please specify

Why do you have dogs? (you can pick more than one reason)

☐ Companion
☐ Control pests (e.g. mice, rats)
☐ For cultural reasons
☐ For kids
☐ Guard dog
☐ Hunting
☐ They needed a home
☐ Other

Please specify

Do you think of your dog as part of your family?

◯ Yes
◯ No
◯ Don't know

Do you care for (e.g. feed) any extra dogs or cats that aren't yours (e.g. pets of relatives or neighbours, homeless strays, feral cats)?

◯ Yes
◯ No

If yes, please tell us about them

Do you have any animals you don't want? (e.g. too old/sick, too many, extra puppies/kittens)

◯ Yes
◯ No

If yes, please tell us about them

Have any of your dogs or cats had puppies or kittens in the last 12 months?

◯ Yes
◯ No
◯ Don't know

If yes - was the litter planned?

◯ Yes, the litter was planned
◯ No, the litter wasn't planned but it was a happy surprise
◯ No, the litter wasn't planned and the litter was an unwelcome surprise

Have you ever had a dog get sick or die from parvo?

◯ yes
◯ no

If yes - please tell us what happened
((How long ago? Did any pets die? How old were they?))

When do you take your dog to the vet?
(Check all that apply)

☐ For regular check-ups
☐ When they are sick (e.g. not eating, vomiting)
☐ For vaccinations (e.g. parvo needle)
☐ When they are injured (e.g. broken leg, hit by car)
☐ For desexing (so no more puppies/kittens)
☐ None of the above
☐ Other

Please specify

Do any of these things stop you taking your animals to the vet?
(Check all that apply)

☐ Cost (too expensive)
☐ Time (e.g. I'm too busy)
☐ Transport (e.g. I can't easily take my pet to the vet because I don't drive)
☐ Uncooperative animal (e.g. my cat is too hard to catch OR my dog is very strong and can be hard to control)
☐ None of the above
☐ Other

Please specify

How often do you spend time interacting with your dog (e.g. playing games, going for walks, sitting on your lap, patting)

◯ Every day
◯ Not every day but more than once a week
◯ Less than once a week but more than once a month
◯ Rarely
◯ Never

How often does your dog go out with you (e.g. on walks, hunting trips, holidays, drives in the car)?

◯ Every day
◯ Not every day but more than once a week
◯ Less than once a week but more than once a month
◯ Rarely
◯ Never

Do any of these things make you want to interact with your dog?
(Check all that apply)

☐ My dog makes me happy
☐ I like sharing stories about my dog (e.g. with friends, family)
☐ Looking after them makes me feel good about myself
☐ My dog help me when I feel stressed or sad
☐ My dog gets me out of the house
☐ My dog helps me feel safe (e.g. at home alone, on walks at night)
☐ None of the above
☐ Other

Please specify

Do any of these things stop you from interacting with your dog?
(Check all that apply)

☐ they are dirty
☐ they have parasites or is otherwise unhealthy
☐ they are too naughty (jumps up, too rough etc)
☐ they are aggressive
☐ they are scared of people, doesn't want to interact
☐ Too busy, not enough time for interacting with pets
☐ None of the above
☐ Other

Please specify

Do you find looking after your dog causes you stress or worry? (e.g. they're very expensive to feed, I worry about my pups getting parvo or getting into fights, they cause me trouble with my neighbour or the council etc.)

◯ Yes, often
◯ Yes, sometimes
◯ No

If yes, what about having animals causes you stress or worry?

Is there anything else you'd like to tell us about your animals?

SECTION 2 - Dogs in the community

Do you think there is a problem with dogs in your community?

◯ Yes
◯ No
◯ Don't know

If yes - please tell us what you think the problems are with dogs in your community

In the last 12 months have you or anyone in this house been
(Check any that apply)

☐ Chased or frightened by a dog
☐ Bitten by a dog
☐ None of the above

Would you like to tell us about what happened?

How often do your dog roam away from your property?

◯ Always
◯ Often
◯ Sometimes
◯ Never
◯ Don't know

Do your animals have any problem behaviours you'd change if you could (e.g. digging holes in the garden, jumping up on people, getting into garbage etc.)

◯ Yes
◯ No

please specify

Is there anything else you'd like to tell us?

SECTION 3 - Dogs and your house

Is the house you live in

◯ Privately owned
◯ Private rental
◯ Community housing (MLAHMC/LALC/MPH etc)
◯ NSW Housing (housing commission)
◯ Other

Please specify

Do any of your animals come inside the house?

◯ Yes
◯ No

If yes, are you able to control which parts of your house your animals can access (e.g. I don't really want the cats in my bedroom but they've figured out how to open the door OR I would keep my dogs out of the kitchen if it had a door or gate)

◯ Yes
◯ No

If yes, which areas can they come into? (check all that apply)

☐ The whole house
☐ Living room
☐ Bedroom/s
☐ Kitchen
☐ Bathroom
☐ Laundry
☐ Other

Where does your dog sleep?

◯ Inside
◯ Outside
◯ Don't know

Please specify (e.g. in bed with me, on the couch, under the house...)

Where do you feed your dog?

◯ Inside
◯ Outside
◯ Don't know

Please specify (e.g. in the kitchen, at the back door...)

Does your property have fences that can keep your dogs in/other dogs out?

◯ Yes
◯ No

27. How often do you leave gates open?

◯ Always
◯ Often
◯ Sometimes
◯ Never
◯ Don't know

Are your pets able to get under your house?

◯ Always
◯ Often
◯ Sometimes
◯ Never
◯ Don't know

Do you keep your dog on a chain?

◯ Always
◯ Often
◯ Sometimes
◯ Never
◯ N/A

If yes, why do you keep your dog on a chain? (Check all that apply)

☐ Because fences and/or gates aren't secure
☐ To stop my dogs from fighting with each other
☐ To stop my dogs breeding
☐ To stop my dogs attacking family members or visitors
☐ Other
